# Quality of life in persons at risk for bipolar disorder: a two year prospective-longitudinal observational cohort study (BipoLife)

**DOI:** 10.1186/s40345-025-00373-y

**Published:** 2025-02-18

**Authors:** Johanna Glaus, Anne Karow, Martin Lambert, Pia Sowada, Kyra Bröckel-Bundt, Christina Berndt, Cathrin Sauer, Georg Juckel, Andreas J. Fallgatter, Andreas Bechdolf, Andreas Reif, Silke Matura, Sarah Kittel-Schneider, Thomas Stamm, Tilo Kircher, Irina Falkenberg, Andreas Jansen, Christoph U. Correll, Paolo Fusar-Poli, Michael Bauer, Andrea Pfennig, Anja Christine Rohenkohl

**Affiliations:** 1 Department of Psychiatry and Psychotherapy, University Medical Center Hamburg-Eppendorf (UKE), Martinistr. 52, 20246 Hamburg, Germany; 2 Department of Psychiatry and Psychotherapy, Faculty of Medicine, TUD Dresden University of Technology, Fetscherstrasse 74, 01307 Dresden, Germany; 3 Department of Psychiatry and Psychotherapy, LWL‐University Hospital Bochum, Ruhr‐University Bochum, Bochum, Germany; 4 Dept. of Psychiatry and Psychotherapy, University Hospital Tübingen, Osianderstr. 24, 72076 Tübingen, Germany; 5 German Center for Mental Health (DZPG), partner site, Tübingen, Germany; 6 Clinic for Psychiatry and Psychotherapy, Charité University Medicine Berlin, Vivantes Klinikum am Urban und Vivantes Klinikum im Friedrichshain, Dieffenbachstr. 1, 10967 Berlin, Germany; 7 Department of Psychiatry, Psychosomatics and Psychotherapy, University Hospital Frankfurt am Main – Goethe University, Heinrich-Hoffmann-Str. 10, 60528 Frankfurt am Main, Germany; 8 Department of Psychiatry, Psychosomatic Medicine and Psychotherapy, University Hospital Würzburg Margarete-Höppel-Platz1, 97080 Würzburg, Germany; 9 Department of Psychiatry and Neurobehavioural Science, University College Cork, Acute Mental Health Unit, Cork University Hospital, Wilton, Cork, T12DC4A Ireland; 10 APC Microbiome Ireland, University College Cork, College Road, Cork, T12 CY82 Ireland; 11 Department of Psychiatry and Psychotherapy, Philipps-Universität Marburg, Rudolf-Bultmann-Strasse 8, 35039 Marburg, Germany; 12 Department of Psychiatry, Psychotherapy and Psychosomatic, Medical School Brandenburg, Neuruppin, Germany; 13 Department of Child and Adolescent Psychiatry, Charité Universitätsmedizin, Berlin, Germany; 14 Department of Psychiatry, The Zucker Hillside Hospital, Northwell Health, Glen Oaks, NY USA; 15 Department of Psychiatry and Molecular Medicine, Donald and Barbara Zucker School of Medicine at Hofstra/Northwell, Hempstead, NY USA; 16 Early Psychosis: Interventions and Clinical-Detection (EPIC) Lab, Department of Psychosis Studies, King’s College London, London, UK; 17 Department of Brain and Behavioral Sciences, University of Pavia, Pavia, Italy; 18 Outreach and Support in South-London (OASIS) Service, South London and Maudlsey (SLaM) NHS Foundation Trust, London, UK; 19 Department of Psychiatry and Psychotherapy, Ludwig-Maximilian-University Munich, Munich, Germany

**Keywords:** Quality of life, Bipolar disorder, Population at risk, Prodromal symptoms, Coping strategies, Life quality, Early recognition, Early intervention, Risk level, Prevention

## Abstract

**Background:**

Improving quality of life (QoL) is important for the treatment of people with bipolar disorder (BD). Early-BipoLife is a German multicentre naturalistic, prospective-longitudinal observational cohort study investigating early recognition and intervention in people at increased risk of developing a BD. This analysis aims to investigate influencing factors and changes in QoL as a basis for the development of early intervention strategies in patients with at risk syndrome for BD.

**Method:**

A cohort of 1086 participants (15–35 years) with at least one risk factor (EPI*bipolar criteria*) for BD was assessed over the course of 2 years. Changes in QoL (WHOQOL-BREF) were evaluated in a mixed model for repeated measures.

**Results:**

Compared to an age-matched comparison group, people at risk for BD showed significant lower QoL in all domains at baseline. The overall QoL of the psychological well-being domain of the WHOQOL-BREF increased over the 2 year study course (*p* < 0.001). The bipolar risk group (EPI*bipolar*) change from baseline divided into (a) decreasing, (b) increasing and (c) constant risk group in the course of 2 years. Baseline risk group assignment was not a significant predictor of change in QoL over 2 years for any of the QoL domains, but participants with an increase in risk over the 2-year course had a significantly smaller gain in QoL than the group with constant risk (*p* = 0.014) or decreasing risk (*p* < 0.001). Higher levels of QoL were associated with a higher self-rated ability to use coping strategies. Moreover, a higher level of functioning (GAF) at baseline was positively correlated with improvement of different QoL domains after 2 years.

**Conclusion:**

Patients with a risk syndrome for BD reported significantly reduced QoL compared to their age-matched comparison group. Risk status monitoring might be beneficial to identify individuals who could profit from an intervention to increase their QoL. Further studies promoting the development of coping strategies for successful self-management could be helpful to improve overall mental health and positively influence QoL.

## Introduction

Bipolar disorders (BD) are among the six most common causes of disability-adjusted life years worldwide and the second one in the group of mental health disorders (WHO 1995). In a survey, bipolar patients stated, that they would rather give up about 40% of their life expectancy, if they would experience a better mental health (Tsevat et al. 2000), demonstrating how severely impacted they are. Therefore, Quality of Life (QoL) has become a crucial role in the care of BD (Murray and Michalak 2012; Morton et al. 2017). First signals of the disorder appear in early adolescence, while the manifestation usually starts between late adolescence and early adulthood (Carlson et al. 2000) resulting in a meta-analytic peak age at onset of 19.5 years, 32% of cases originate before 25 years (Solmi et al. 2022).

The World Health Organization (WHO) illustrates QoL as an “individuals’ perception of their position in life in the context of the culture and value systems in which they live and in relation to their goals, expectations, standards and concerns” (WHO 1995). To evaluate QoL using the WHOQOL-BREF, four subdomains are assessed: psychological well-being, physical health, social relationships, and environmental well-being (WHO 1995). This patient-reported outcome allows the patient to be in the centre of determining the effectiveness of a treatment intervention. It facilitates the examination of patient preferences and can support the comparison of well-being between different conditions (Michalak et al. 2005). Improvement in QoL has been selected as the most important outcome for treatment of bipolar illness by patients and clinicians (Murray et al. 2017). Evidence has shown that there could be bidirectional effects in the interaction between symptoms and QoL. For instance, there is a positive impact of QoL on reducing symptoms (Morton et al. 2018). A related concept to QoL is resilience, which is additionally associated with a better overall QoL in bipolar patients (Lee et al. 2017). Resources and self-management skills may have a positive influence on resilience (Pfennig et al. 2020). Self-management as a relevant coping skill describes the individual’s ability to manage their health and maintain QoL through learning and utilizing skills (Jones et al. 2011). It empowers the patient to be in control of their mental health progression instead of being at the mercy of external stimuli and internal predispositions. The role of the patient shifts to be the expert or at least the partner of the physician in treatment (Draper 2013). Fortunately, a variety of self-management-focused interventions are already available, making it valuable to review the evidence on the effectiveness of self-management (Janney et al. 2014). Some applications are even designed for prevention (Karasouli and Adams 2014).

To examine the right moment to implement prevention strategies for people at risk of developing a BD, more detailed research of the prodromal phase is beneficial. The bipolar prodrome consists of precursor symptoms, functional impairments and further psychiatric diagnoses which can be noted months or even years forgoing the onset (Conroy et al. 2018). Precursors can be anxiety disorders, mood liability, mood swings, cyclothymia, subthreshold manic and hypomanic episodes, panic anxiety, subsyndromal depression, early age of onset of depressive episodes and suicidal behaviour (Conroy et al. 2018; Faedda et al. 2015, 2019; Howes and Falkenberg 2011). By collecting risk factors (e.g. family history of BD) or the previously mentioned symptoms the risk to develop a BD can be estimated.

In summary, the interest in QoL is growing in the field of BD. It is a powerful multidimensional construct that places patients’ needs and interests at the center of treatment evaluation, thereby helping to identify grievances and areas where improvement is most needed from the patient’s perspective. Even though patients with BD declared that QoL is their primary treatment goal (Jones et al. 2013), there is, best to our knowledge, almost no research on overall QoL of people at risk including the differentiation between the domains (physical health, psychological well-being, social relations, environment) and how their QoL is impacted over a 2 year course. Research to identify patients’ well-being already diagnosed with BD has been made, but there is a lack of knowledge on how people at risk for BD are impacted and how we can improve preventive strategies with a focus on QoL.

The purpose of this analysis is to shed light on the QoL of people at risk of BD. To suggest new prevention approaches, influences on the course of QoL are further investigated. The relation between the course of QoL and the risk score of a development of a BD at baseline and the risk group transformation during the 2-year period could lead to preventive recommendations. Insight into the impact of self-management skills on QoL could similarly lead to beneficial recommendations.

Our analyses are based on the following hypotheses: (1) Quality of Life of people at risk of developing a BD is significantly lower compared to their norm peers. (2) QoL changes over the 2 year observation period, while a decrease of the risk status is associated with an increase in QoL. (3) A high level of self-management skills at baseline is positively associated with an increase in QoL.

## Methods

### Context

Early-BipoLife A1 is part of the German Research Consortium BipoLife (Ritter et al. 2016) with a focus on bipolar disorders (Pfennig et al. 2020). It is a Germany-wide multicentre study in which 10 universities and teaching hospitals with screening and treatment programmes for BD participated. The BipoLife project was funded by the German Ministry of Education and Research (BMBF, Grant number: 01EE1404A). Extended information of the overall study design has been published within the study protocol (Pfennig et al. 2020). The overall aim of the A1 project is improving early recognition and intervention in people at‑risk of developing a BD (Pfennig et al. 2020).

### Study design

Early-BipoLife is a naturalistic, prospective-longitudinal observational cohort study. Recruitment took place in early recognition centres and if not already implemented, individualized treatment as usual was provided. The participants were seen at baseline (BL), and 4 follow-up assessments (FU1-FU4) during a course of at least 2 years from July 2015 until September 2018. In order to map the course in QoL, data from the following assessments in which QoL was evaluated: baseline (BL), follow up 2 after 12 months (FU2) and follow up 4 after 24 months (FU4) was included. FU1 took place after 6 months and FU3 after 18 months and were both performed as telephone-interviews without assessing QoL data. The overall time of an examination was 3–7 h. The interviewers conducted a comprehensive standardizes face-to-face diagnostic interview and were trained 2 days centrally and later supervised and retrained by their principal investigator locally. The examinators were physicians and psychologists, not blinded for the bipolar risk status. The ethics committee of the Technical University Dresden and all local ethics committees approved the study. Only participants who gave their written informed consent were included. For minors <18 years it was a requirement to obtain their informed assent and have one of their caretakers confirm the consent (Pfennig et al. 2020).

All participants received state of the art counselling and treatment tailored to their individual needs, selected by the expertise and experience of their local study centre as requested in a naturalistic design (Pfennig et al. 2020).

### Participants

Participants aged 15–35 years with at least one risk factor for the development of a BD were recruited at the German hospitals in Berlin, Bochum, Dresden, Frankfurt/Main, Hamburg, Marburg, Brandenburg/Neuruppin and Tübingen (Pfennig et al. 2020). 1229 participants from these sites across Germany were included in the longitudinal study. The following risk factors compose the *inclusion criteria*: family history of BD, (sub)threshold affective symptomatology, hypomanic/mood swings, disturbances of circadian rhythm/sleep, ADHD diagnosis and depressive/dysthymic disorders (Pfennig et al. 2020). *Exclusion criteria* were a diagnosis of a BD, schizoaffective disorder, schizophrenia or a diagnosis of anxiety, obsessive–compulsive or substance dependence disorder. A limited ability to comprehend the study or acute suicidality also led to exclusion. The first data on 2-year findings were published recently (Martini et al. 2023). From the overall 1229 participants, 1038 filled in the QoL self-report questionnaire at baseline (BL) and were included in the present analysis as the self-rating and participation in the study were voluntary.

For a comparison with an appropriate norm, the data from the Hawthorne et al. (2006) data set was used. The sample was age matched (20–29 years) and includes a broad range of health conditions from full health to terminal illness (Hawthorne et al. 2006).

### Measures

Clinical and socio-demographic data throughout the study were assessed using the Structured Clinical Interview (Wittchen et al. 1997; Fydrich et al. 1997) together with instruments that address symptoms of psychotic prodrome (Ising et al. 2012; Miller et al. 2003; Schultze-Lutter et al. 2012) and psychiatric treatment, physical illness and substance use utilizing case report forms (CRF).

The Early Phase Inventory for Bipolar Disorders (EPI*bipolar*) is a semi-structured interview and was developed by Pfennig and Leopold et al. in 2012 to capture specifically the risk status regarding an onset of bipolar illness (Leopold et al. 2012). The EPI*bipolar* was used to address the course of disease in terms of change of severity of the risk status. Risk factor categories and information of the patient’s history were determined through a systematic review of the literature and clinical experience and form the basis for the classification into three risk categories (no risk, low risk, high risk). Included criteria: e.g. family history of BD, subthreshold depressive and (hypo-)manic symptoms, substance misuse, a diagnosis of ADHD or behavioural problems/conduct disorder, pronounced creativity, critical life events, changes in sleep/circadian rhythm, mood swings or increased affective lability, fearfulness/anxiety, dissociative symptoms, and impairment in psychosocial functioning (Pfennig et al. 2020). For the risk quantification these risk factors are listed, weighted and assigned to one of the three final groups depending on fulfilment of the aforementioned risk factors (Leopold et al. 2012).

The WHOQOL-BREF as a Patient Reported Outcome (PRO) instrument was used to assess the global health status of patients independently of a disease. This questionnaire consists of 26 items in total (Angermeyer et al. 2000), which are assigned to four domains: physical health (energy level and the ability to perform daily activities), psychological well-being (emotional and cognitive health), social relationships (personal connections such as family and friends) and environment (safety and access to resources). All items are answered on a five-point Likert scale. There are four types of a five-point Likert interval scale, which reflect intensity, capacity, frequency and evaluation, and one of these was attached to each item. Items inquire ‘how much’, ‘how completely’, ‘how often’, ‘how good’ or ‘how satisfied’ the respondent felt in the last 2 weeks. Domain scores were built as sum scores and transformed on a 0 to 100 scale to enable comparisons between domains composed of unequal numbers of items. The higher the score the better the self-perceived QoL (WHOQOL-Group 1998). Norm data have been published for all domains by Hawthorne et al. (Aigner et al. 2006) and are provided in Table 3.

The FERUS (Fragebogen zur Erfassung von Ressourcen und Selbstmanagementfähigkeiten, in English: Questionnaire for Assessing Resources and Self-Management Skills) measures health-related resources and self-management skills, comprising of 66 items reflecting 7 scales (motivation to change, self-observation, active and passive coping, self-efficacy, self-verbalisation, hope and social support) and a total score (Jack 2001). This total score represents the overall self-management ability and was included in the present analyses. The FERUS is answered on an agreement scale from 1 "not true" to 5 "very true"; scores were built as sum scores. According to the FERUS, the higher value, the more pronounced the self-management resource is.

### Statistics

#### Course of QoL

In order to get better insight into QoL in the population of individuals at risk of bipolar disorder, the WHOQOL-BREF was first evaluated via descriptive statistics [mean (M), standard deviation (SD)] for the 4 subdomains of QoL (physical health, psychological well-being, social relations and environment). A comparison with a norm population (Hawthorne et al. 2006) was calculated with a T-test for one sample, reporting the test statistics T, *df* and *p* for significant results at baseline and after 2 years. The changes in QoL over the 2 year-period were evaluated in a mixed model for repeated measures (MMRM) and are described below.

#### Predictors for the course of QoL

Beside the changes in QoL, the predictors were addressed as well in the MMRM for each of the four domains separately covered by the WHOQOL-BREF. The QoL measures at follow-up times (12 months (FU2) and 24 months (FU4) after baseline) were considered as repeated measures, the patients as the random effect, time as fixed effects, and the baseline (BL) values of the dependent variables as well as age and sex as covariate. Outcomes were changes from baseline in QoL scores of the WHOQOL-BREF (dependent variable). As potential *sociodemographic predictors* of QoL a positive family history for mental illness (yes/no) and employment status (no employment, paid education, part time or less, full time) were included as independent variables, because of their relevance as risk factor and predictor for poor outcomes in mental disorders (Solmi et al. 2023). Regarding *clinical characteristics*, the following variables have been taken into account as independent variables additionally, derived from the literature described above: substance disorder (easy/medium/severe), global assessment of functioning score (GAF, continuous), current psychiatric treatment (yes—outpatient, yes—partly inpatient, yes—inpatient, no), ever received psychiatric treatment (no consultation, consultation or short treatment, continuous treatment for months, continuous treatment over years), current medication (yes/ no). The risk factor for developing a BD was imaged via the EPI*bipolar* (no risk, low risk, high risk). To reflect the change in risk score from baseline to FU4, a new variable was created taking the change in EPI*bipolar* over the 2-year period into account (increasing risk, decreasing risk, constant risk). Both variables regarding the risk factor were included as predictor variables (independent variable) as well. In addition, the *coping* total sum scale of the FERUS “self-management”, assessed at baseline, was entered in the MMRM model as potential predictor too. Baseline values were used as covariates to minimize the variance. The main effects (F), significance levels (*p*) and 95% confidence intervals (CI) are reported. In addition, correlations (Pearson product-moment correlation coefficient, *r*) were calculated between the self-management scale and all subdomains of the WHOQOL-BREF at baseline and FU4 to map the relationship between coping resources and QoL in Early-Bipolife. The level of significance was set at *p* < 0.05, two sided. The data were analysed using IBM SPSS Statistics (Version 27) (Version 2020).

## Results

### Sample characteristics

The sample of the present analysis consists of 1038 participating adolescents and adults who completed the self-report to assess QoL at baseline. The mean age was 24 years (range: 15–35 years). Overall, more women (55.3%) than men participated. For the risk score, data from *N* = 1043 were available at baseline. According to the inclusion criteria people with at least one proposed risk factor for the development of bipolar disorder were included. Out of these according to EPI*bipolar* criteria at baseline, 21% were in the group that showed no risk for developing BD (help seeking persons). 43% were at low risk and 32% were at high risk of developing BD prospectively; data from 4% are missing. Most participants in FU4 had a consistent risk over the 2-year period (64%). 24% had a decreasing risk-score and 12% had an increasing risk-status (Table 1).

**Table 1:** Sample characteristics

Sociodemographic and clinical characteristics at baseline		*N*	*M* (SD)/percentage*
Age (years)		1038	24.35 (4.49)
Sex	Female	574	55.3
	Male	464	44.7
Highest education**	No degree	6	0.6
	9th grade diploma	46	4.4
	10th grade diploma	192	18.5
	12th grade diploma	739	71.2
	In education	45	4.3
	Other	3	0.3
Employment	No employment	484	46.6
	Paid education	88	8.5
	Part time or less	291	28.0
	Full time	159	15.3
Currently receiving medication?	Yes	667	64.3
	No	363	35.0
Currently receiving psychiatric treatment?	Yes—outpatient	272	26.2
	Yes—partly inpatient	102	9.8
	Yes—inpatient	261	25.1
	No	396	38.2
Ever received psychiatric treatment?	No consultation	358	34.5
	Consultation or short treatment	251	24.2
	Continuous treatment for months	148	14.3
	Continuous treatment over years	246	23.7
Mentally ill relatives?	Yes	758	73.0
	No	218	21.0
Substance disorder (SKID-I)	Mild	66	52.0
	Medium	52	40.9
	Severe	9	7.1
Risk-group for bipolar diagnosis (EPI*bipolar*)	No risk	222	21.4
	Low risk	465	44.8
	High risk	342	32.9
Risk-group for bipolar diagnosis (EPI*bipolar*) change from Baseline to FU4	Increasing risk	56	12.3
	Decreasing risk	98	21.6
	Constant risk	300	66.1
Global assessment of functioning (GAF)		1007	64.46 (18.08)

Coping (FERUS) at baseline			
Self-management		1035	134.70 (30.31)

*Indication of the valid percentages **this variable was later removed from the model due to too small numbers in subcategories, *N* sample size, *M* mean, *SD* standard deviation, *SKID-I* structured clinical interview for DSM-I

Drop-out rates for this sample are displayed in Table 2. 109 of the participants (9.5%) were no longer interested in taking part in the study. During the study period, 22 participants (2.0%) were diagnosed with conversion to BD and were excluded from the study.

**Table 2:** Drop-out rates and reasons

*N* = 1038	Drop-out		
	Baseline (BL)	Before FU2	Before FU4
Conversion to bipolar disorder	1	9	12
Conversion to diagnosis of exclusion		3	1
SAE		1	
No interest	10	68	17
*N* drop-out	11 (1.06%)	81 (7.89%)	30 (3.17%)

	*N* BL	*N* FU2	*N* FU4
Participants	1027	946	929

*FU* follow-up, *N* = sample size, *SEA* serious adverse event

### Course of quality of life

Compared to the age-matched control group, the risk sample shows significantly lower QoL on all domains of the WHOQOL-Bref at baseline (physical health (T = −42.96, *df* = 1037, *p* = < 0.001), psychological well-being (T = −35.15, *df* = 1037, *p* = < 0.001), social relations (T = −22.97, *df* = 1037, *p* = < 0.001) and environment (T = −14.00, *df* = 1037, *p* = < 0.001). The results can be similarly replicated across the entire sample for the time point after 2 years; only the environment scale clinically reaches the normal value.

For the course in QoL domains covered by the WHOQOL-BREF, the mean scores increase on all domains (see Table 3). A clinically relevant gain in QoL is particularly evident from BL to FU2, but significant changes can only be reported for the domain psychological well-being (BL-FU2: *p* < 0.001).

**Table 3:** Course of quality of life

WHOQOL-BREF (domains)	Norm data M (SD)	Baseline (BL) M (SD)	12 months (FU2) M (SD)	24 months (FU4) M (SD)
Physical health	85.4 (10.9)	60.27 (18.85)	67.83 (16.02)	67.84 (16.76)
Psychological well-being	71.4 (17.5)	47.11 (22.26)	54.73 (20.16)	57.45 (19.92)
Social relations	72.9 (18.8)	56.62 (22.85)	60.67 (19.30)	63.51 (20.67)
Environment	74.3 (14.0)	67.38 (15.92)	71.82 (13.83)	73.59 (13.52)

*WHOQOL-BREF* World Health Organization Quality of Life Instrument: short form, *M* mean, *SD* standard deviation

**Fig. 1:**
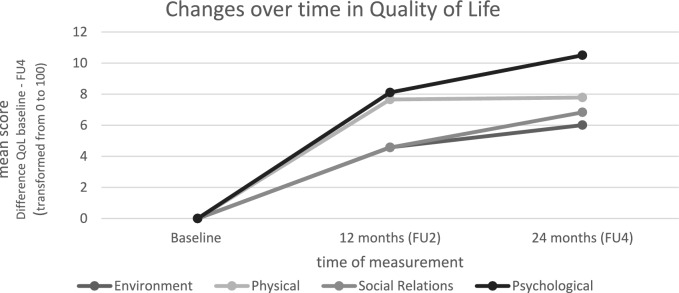
Changes over time in QoL. *Mean values of the change in Quality of Life from baseline to Follow up 4 (FU4)

Figure 1 shows the change in QoL over the course from baseline to FU2 to Fu4.

### Factors influencing the course of quality of life

In the result report on the MMRMS of the subscales, a distinction is made between socio-demographic and clinical variables as well as coping (self-management). *Sociodemographic variables*: The variables highest education and employment status were excluded from the model because subgroups were too small. Regarding a positive family history for mental illness no significant results can be reported. Age and sex were included as covariates. *Clinical variables*: The level of functioning at baseline, as measured by the GAF, had a significant impact on the progression of QoL in several domains of the WHOQOL-Bref, such as psychological well-being (F = 9.81;* p* = 0.002) and environment (F = 8.44; *p* = 0.005). The higher the functional level at baseline, the greater the gain in QoL on these domains. Baseline risk group assignment (EPI*bipolar*) was not a significant predictor of change in QoL over 2 years for any of the QoL domains. Looking at the changes in the risk groups (Baseline to FU4), however, we found that those with increasing risk have a significantly smaller gain in QoL than the group at the constant risk (*p* = 0.014) or with decreasing risk (*p* = 0.002) on the psychological well-being domain (F = 8.92; *p* < 0.001). The group, which decreases from baseline to FU4 in their risk assignment, shows the greatest gain in QoL regarding psychological well-being. *Coping* as assessed by the FERUS scale only self-management had a significant influence on the QoL scale environment (F = 4.46; *p* = 0.039). The higher the self-management resource at baseline the smaller the gain in the environment scale (−0.164). The correlation between self-management and environment was positive and significant at all three measurement points. Those with high resources in self-management also had a high score in environment at baseline.

### Correlations between coping resources and quality of life

The correlation coefficients between the assessed coping resource self-management and QoL are shown in Table 4 below. Results show that the correlations are moderate to strong (*r* = 0.438–0.714) and significant at the 0.01 level (2-sided) for all subdomains of the WHOQOL-BREF.

**Table 4:** Correlation between self-management and Quality of Life

	WHOQOL			
	Physical health	Psychological well-being	Social relations	Environment
	BL	BL	BL	BL
FERUS BL self-management	r = 0.539**	r = 0.714**	r = 0.438**	r = 0497**

**Significant at the 0.01 level (2-sided)

## Discussion

Our results indicate that people at risk of developing a BD from age 15–35 have a significantly worse QoL than an age-matched comparison group. After receiving consultation from an early detection centre in Germany an increase in the psychological-wellbeing domain of QoL was observed. The other domains (physical health, social relationships, environment) showed no significant improvement during the course of 2 years. Relevant variables influencing QoL over the course of 2 years were the level of functioning at baseline (assessed with the GAF), self-management skills at baseline. People with an increasing risk over the 2 years had a significantly lower gain of QoL in comparison to the people with constant or decreasing risk of developing BD. The EPI*bipolar* risk status (no risk, low risk, high risk) at baseline was not a predictor for change in QoL after 2 years.

Measuring QoL is a simple tool to get a more holistic picture of the patients’ well-being (Hertz-Palmor and Gothelf 2022). It can facilitate the identification of youth with a low well-being even when psychiatric symptoms are not visible (Hertz-Palmor and Gothelf 2022). As previously highlighted, QoL was selected as the most important treatment outcome by bipolar patients (Murray et al. 2017). There is a need for improvement of the support and therapy services provided by our social and healthcare system, because the results of this analysis show that QoL of youth at risk for the development of a BD is significantly lower compared to their peers.

Although this was not an RCT study and therefore no causal correlations can be inferred, our results suggest that seeking contact to an Early Detection Centre is connected to an improvement in the psychological-wellbeing domain of QoL. The observed trend underscores the potential value of early professional support for young people at risk of BD. We encourage further research with controlled designs to investigate these effects in more detail. The psychological domain may exhibit the highest sensitivity to interventions. Although improvements were observed across all subdomains, it is plausible that a longer observation period could yield statistically significant changes in other domains. Notably, shifts in social structures and behavioral patterns might only manifest over extended periods, potentially requiring several years to become detectable. Bipolar disorder is primarily characterized by emotional distress, which may explain why the physical domain is less strongly affected. Additionally, the cohort largely consisted of adolescents and young adults who often lack the decision-making authority or financial resources to significantly alter their environment. Consequently, the interventions provided by an Early Detection Center likely had a limited impact on the environmental domain.

Interestingly, the risk level status at baseline was not a predictor of QoL progression over the next 2 years, but its´ change over time was. To facilitate a QoL enhancement, a stabilization or reduction of the risk-score may be sufficient, even with a high-risk status at the beginning. Additionally having a low-risk status does not automatically lead to a better psychological wellbeing in the future. Supporting a decreasing or stable level of risk can have a positive impact on psychological wellbeing regardless of their initial status. Our findings suggest, that enhancing the level of functioning, while preventing an increase of the risk level could benefit the QoL of youth at risk of developing a BD.

As mentioned, bidirectional effects between QoL and the impact on lowering symptoms has previous been reported (Morton et al. 2018). As written in the German guidelines for the treatment of BD, the current approaches for primary prevention are aimed at teaching coping strategies and stress reduction Pfennig et al. 2013. Developing an effective intervention for people at this vulnerable stage could be of great importance. In this study, the exact factors that influenced the risk status change were not recorded or controlled, as this was not a randomised-controlled trial (RCT). But it was shown that coping skills at baseline and QoL at baseline had a significant correlation. Although coping skills at baseline do not influence the risk status change, gaining coping strategies might still have a positive impact, but as the self-management skills have not been assessed a second time, we cannot draw valid conclusions about that. Further studies to find out which strategies are beneficial in influencing the risk score would be advisable and should include coping skills. As an indicator of who could benefit from treatment, it can be deduced from our results that everyone who seeks to go to an early recognition centre could benefit from an intervention. As the whole sample was impaired in their self-perceived QoL and included people with only one risk factor but no risk status for the development of BD and showed an overall increase in QoL over the 2 year observations period, one could conclude that every help seeking person might profit from support services. The moment of going to a consultation might come after a perceived impairment of their QoL, making this point a good indicator for the need for treatment. Group therapy with peer to peer knowledge sharing could be implemented and useful online tools could provide a broad and economically sensible service strategy. As risk monitoring can positively influence the psychological QoL enhancement, an implementation into guidelines for diagnostics and treatment of BD (Pfennig et al. 2013) should be considered.

In order to obtain a broader assessment of QoL changes in the at-risk population, a combination of risk scores and resilience skills might help to identify risk progression groups. These groups could, for example, better reflect individual progression and therefore indicate which individuals could potentially benefit from an intervention to enhance their QoL. An aftercare group, which is regularly connected following the diagnosis of a risk status, should include patient skills, resources and strengthening coping strategies. Just as the shift from third-party reporting to patients’ self-reporting of their own QoL took place a few years ago, a similar development should also happen in other areas. Emphasizing the individual’s part in the course of their own life gives patients back the responsibility and ability not to be determined by clinical risk factors.

## Limitations and outline

The advantages and limitations of the Early-BipoLife Study have been previously published (Pfennig et al. 2020). The sample size of 1086 participants and the originality of the study design (at risk population, duration of 24 months) appear to be the main advantages for an informative analysis and transferability of the results. The low conversion rate of 2%, even though 43% of the participants were classified at low risk and 32% at high risk, might be a signal that the duration period of 2 years should be extended in further studies. The study cohort is characterized by a relatively high level of education and a predominance of unemployment, which we attribute to the age demographic of the at-risk population and the geographical location of the Early Detection Centers. These centers are associated with university hospitals and are situated in Germany’s largest cities, potentially influencing the cohort’s composition.

To our knowledge, this is the first study to monitor the quality of life of people at risk of developing BD between the ages of 15 and 35 over a 2-year period, which makes it difficult to compare and place the results in the context of previous research.

Further studies that observe the influence of strengthening coping strategies on the change in risk status would be valuable and promising. Our findings suggest that the psychological wellbeing domain increases during a 2 year long treatment phase for people at risk. As this study was a naturalistic study and not a RCT, we can not definitively establish a causality between the contact period and the gain in QoL. It could be useful to study further what factors exactly impacted the enhancement to design a helpful intervention program. Since this multicenter study focuses on the general impact of engagement with an early detection center, individual therapeutic approaches were not analyzed. Exploring this in future studies would be highly valuable.

## Conclusion

In summary, adolescents with risk factors for BD have a significantly lower QoL than their peers and might profit from a gain in psychological wellbeing through consulting an early detection center. Both, people with a low- and high-risk profile might gain QoL through stabilization or reduction of their risk status. Monitoring the progression of risk status is key and could be offered to all patients. A risk status increase could function as a warning sign for a deterioration in mental health and therefore demonstrate the need for an intervention. Additionally, it may be a useful predictor for the increase in QoL. A high level of functioning and self-management skills is positively associated with improvements in QoL. As part of therapy, developing coping strategies could be helpful in improving mental health.

There is a need for further research to understand the QoL of youth at risk for developing a BD, how to support them efficiently and if and how the manifestation of the bipolar disease could be prevented.

## Data Availability

The data sets analysed during this study are not publicly available because participants did not agree to share their clinical data as an open source. They are, however, available from the corresponding author on reasonable request.

## Abbreviations

BAR citeria: Bipolar at risk criteria
EPIbipolar: Early phase inventory of bipolar disorders
FERUS: Fragebogen zur Erfassung von Ressourcen und Selbstmanagementfähigkeiten
FU: Follow up
M: Mean
MMRM: Mixed model for repeated measures
PRO: Patient reported outcome
QoL: Quality of life
SD: Standard deviation
TAU: Treatment as usual
WHOQOL-BREF: World Health Organization Quality of Life-Brief Version
